# Silver nanoparticle-loaded microgel-based etalons for H_2_O_2_ sensing†

**DOI:** 10.1039/c8ra02215a

**Published:** 2018-04-24

**Authors:** Tong Shu, Qiming Shen, Yu Wan, Wei Zhang, Lei Su, Xueji Zhang, Michael J. Serpe

**Affiliations:** Beijing Key Laboratory for Bioengineering and Sensing Technology, Research Center for Bioengineering and Sensing Technology, School of Chemistry and Biological Engineering, University of Science and Technology Beijing Beijing 100083 P. R. China; Department of Chemistry, University of Alberta Edmonton Alberta Canada T6G 2G2 michael.serpe@ualberta.ca +1 780 492 8231 +1 780 492 5778

## Abstract

Silver nanoparticles (AgNPs) were generated inside the network structure of poly(*N*-isopropylacrylamide)-*co*-acrylic acid (pNIPAm-*co*-AAc) microgels that were sandwiched between two thin Au layers (15 nm) of an etalon. This was done by introducing Ag^+^ to the etalons composed of deprotonated microgels, followed by its subsequent reduction with NaBH_4_. The resultant microgels were collected and then characterized by transmission electron microscopy (TEM), Fourier transform infrared spectroscopy (FTIR) and X-ray photoelectron spectroscopy (XPS), verifying the loading of AgNPs with relatively uniform diameter (5–7 nm) within the microgels. Furthermore, the optical properties of the resultant etalons and their response to H_2_O_2_ were evaluated by reflectance spectroscopy. Specifically, upon the addition of H_2_O_2_, the AgNP-loaded etalons exhibited both a red shift in the position of the reflectance peaks and an increase in reflected wavelength intensity. We hypothesize that the dual signal response of the devices was a result of oxidative decomposition of the AgNPs, enabling the microgels to swell and for more light to be reflected (due to the loss of the light absorbing AgNPs). Finally, we showed that the AgNPs could be regenerated in the used etalons multiple times without a loss in performance. This work provides a cost-effective means to detect H_2_O_2_, which could be modified to sense a variety of other species of physiological and environmental importance through rationally loading other functional nanomaterials.

## Abstract 2

Silver nanoparticle (AgNP)-loaded poly(*N*-isopropylacrylamide)-*co*-acrylic acid (pNIPAm-*co*-AAc)-based microgels were generated and used to make etalons. The etalons were shown to exhibit optical properties that depended on the concentration of H_2_O_2_ in solution.

## Graphical abstract

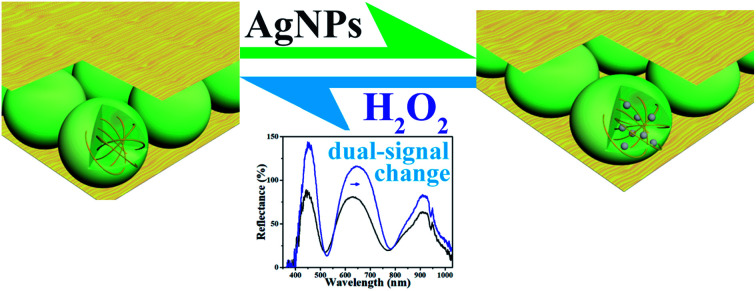

## Introduction

Photonic crystals have attracted considerable interest due to their low cost, ease of signal readout, simple operation, and their ability to be modified to detect multiple analytes.^1–4^ The Serpe Group pioneered the development of poly(*N*-isopropylacrylamide) (pNIPAm) microgel-based etalons in 2010.^5,6^ Since then, they have been shown to exhibit visual color that can be made to depend on the presence of certain species, or the state of a system, *e.g.*, temperature, pH,^7^ Cu^2+^,^8^ CO_2_,^9^ H_2_O_2_,^4^ proteins,^10^ and DNA.^11^ Typically, etalons are constructed by sandwiching a layer of pNIPAm microgels between two thin Au layers.^12^ At a fixed observation angle, the position of the peaks in a reflectance spectrum (and hence the device color) depends primarily on the distance between the two gold layers.^6^ The ability of the etalons to change color is derived from the responsivity of the microgel layer. Importantly, modification of microgels with functional groups, *e.g.*, 4-vinylpyridine, can allow the etalons to respond to specific species.^9^

The past three decades have witnessed an incredible increase in the development of nanomaterials,^13–16^ due to their remarkable size- and shape-dependent electronic, magnetic and optical properties (*i.e.*, the quantum confinement effect) as well as their ability to be used in various applications, *e.g.*, sensors.^17,18^ To generate nanoparticles with narrow size distributions, ligands that yield repulsive forces between nanoparticles to control aggregation are often used.^19^ Of importance for this investigation are pNIPAm-based microgels, which have emerged as a novel and promising nanoparticle stabilizer.^20^ That is, the interior of microgels can serve as a microreactor for generating nanoparticles. For instance, Shi *et al.* synthesized Au nanoparticles inside pNIPAm-*co*-methacrylic acid microgels *via* an *in situ* reduction reaction.^21^ In a previous study, we showed that pNIPAm-*co*-acrylic acid (pNIPAm-*co*-AAc) microgels could be used as a scaffold for the *in situ* generation of silver nanoparticles (AgNPs), which could subsequently be used to detect H_2_O_2_ in solution.^22^

As a reactive oxygen species (ROS), H_2_O_2_ is generated from almost all biological oxidative cycle reactions.^23^ H_2_O_2_ also plays an essential role in modulating a number of physiological processes. For instance, the homeostasis of H_2_O_2_ possesses significant impacts on cell proliferation, death, and signal transduction.^24^ On the other hand, excessive amounts of H_2_O_2_ in the human body can generate irreversible oxidative damage, resulting in accelerated aging,^25^ neurodegeneration,^26^ DNA damage,^27^ and cancer.^28^ Thus, it is essential to continuously develop new and improved tools to quantify H_2_O_2_. In this investigation, we show that microgels in etalons can be used as a scaffold for the generation and stabilization of AgNPs, which we show can be used for generating a “dual” optical response to H_2_O_2_. Specifically, we show that an *in situ* synthetic route for generating AgNP-loaded pNIPAm-*co*-AAc hybrid microgels in etalons could be achieved by first exposing deprotonated pNIPAm-*co*-AAc microgels to Ag^+^. The negative charges on the deprotonated AAc groups allowed the microgels to strongly interact, and become enriched, with Ag^+^. AgNPs with narrow size distribution could then be obtained by performing the reduction at low temperature. The resultant AgNP-loaded etalons were shown to exhibit color due to the structure of the etalon and the absorbance of light by the AgNPs. We hypothesize that the color of the devices changed upon addition of H_2_O_2_ due to the degradation of the AgNPs, which causes a red-shift in the reflectance peaks from the etalon and an increase in the intensity of the reflectance peaks. To this end, we investigated the response of the AgNP-loaded etalon to various concentrations of H_2_O_2_. The dual optical signal change of the etalon can then be used to detect H_2_O_2_, while also providing the possibility for correcting for interferants in solution.^5–8^ We go on to show that the sensing performance of the AgNP-loaded etalon could be fully restored by a repeated exposure to Ag^+^ and subsequent AgNPs generation.

## Experimental section

### Materials and methods

NIPAm was purchased from TCI (Portland, OR) and purified by recrystallization from hexanes (EMD, Gibbstown, OR). *N*,*N*-Methylenebisacrylamide (BIS), acrylic acid (AAc), ammonium persulfate (APS), silver nitrate (AgNO_3_), sodium hydroxide (NaOH), hydrogen chloride (HCl) and sodium borohydride (NaBH_4_) were purchased from Sigma-Aldrich (Oakville, ON). Microgels were lyophilized using a VirTis bench-top K-manifold Freeze Dryer (Stone Ridge, New York). Deionized (DI) water with a resistivity of 18.2 MΩ cm obtained from a Milli-Q Plus system (Billerica, MA), was utilized in this study.

### Characterization

A Hitachi H-7650 transmission electron microscope (TEM, Japan) with an accelerating voltage of 200 kV was utilized to obtain TEM images. X-Ray Photoelectron Spectroscopy (XPS) was performed on a Kratos AXIS Ultra spectrometer equipped with a monochromated Al Kα (*hν* = 1486.6 eV) X-ray source (Kratos Analytical, Manchester, UK). Nicolet Magna 750 FTIR Spectrometer and Nic-Plan FTIR Microscope (Nicolet, USA) with pure KBr as the background was applied to record Fourier transform infrared (FTIR) spectra (400–4000 cm^−1^). The microgel samples for TEM, XPS and FTIR characterization were prepared using the procedure described below for generating AgNPs in the etalons with two modifications. First, to obtain sufficient sample for analysis we didn't wash the slide after microgel painting, which yielded a thick microgel layer for AgNP generation. Second, the etalon's top Au layer was not deposited on the microgels, which allowed for their easy removal from the surface while avoiding potential contamination. Reflectance measurements were conducted using a USB2000+ spectrophotometer, an HL-2000-FHSA tungsten light source, and an R400-7-VISNIR optical fiber reflectance probe, all from Ocean Optics (Dunedin, FL). The spectra were recorded using Ocean Optics Spectra Suite spectroscopy software over a wavelength range of 400–1000 nm.

### Synthesis of pNIPAm-*co*-AAc microgels

pNIPAm-*co*-AAc microgels were synthesized *via* surfactant-free, free radical precipitation polymerization, according to an established protocol.^22,29–33^ The monomer, NIPAm (10.54 mmol), and the crosslinker, BIS (0.703 mmol), were fully dissolved in water (99 mL) with stirring in a beaker for 1 h. The mixture was then filtered through a 0.2 μm filter affixed to a 20 mL syringe into a 250 mL, 3-necked round bottom flask. The flask was then equipped with a thermometer, a condenser/N_2_ inlet/outlet, and a stir bar. The monomer solution was purged with N_2_ gas for ∼1 h while stirring and heating to 70 °C. AAc (2.812 mmol) and APS (0.046 g in 1.0 mL water) was then added to the pre-heated solution, respectively. The reaction continued for 4 h. After cooling down, the turbid solution was filtered through glass wool to remove any large aggregates. The coagulum was rinsed and the collected liquid was diluted to 100 mL. Aliquots of the microgel solution (33 mL) were centrifuged at a speed of 10 000 relative centrifugal force (rcf) at 20 °C for 45 min. The microgels were isolated and redispersed to their original volume (∼33 mL) with water. This centrifugation/resuspension procedure was repeated 6 times. Finally, all of the centrifuged microgels were combined into one tube and diluted to 30 mL with water for storage.

### Etalon preparation

Etalons were fabricated using the techniques that were detailed in previous studies.^4^ Typically, 25 × 25 mm microscope coverslips (Fisher's Finest, Ottawa, ON) were washed with ethanol and dried with N_2_ gas and coated with 2 nm Cr and then 15 nm Au *via* thermal evaporation at rates of 1 and 0.1 Å s^−1^, respectively (Torr International Inc., thermal evaporation system, Model THEUPG, New Windsor, NY). The Cr/Au coating was annealed in a Thermolyne muffle furnace at 250 °C for 3 h and allowed to cool. Then, aliquots (40 μL) of concentrated microgels were added to the annealed Au-coated glass coverslips and spread out to coat the whole surface. The deposited microgels were dried on a hot plate set at 30 °C for 30 min. The excess free microgels not directly adhered to the Au layer were rinsed away with DI water. The samples were then soaked in DI water overnight at 30 °C. The slides were then rinsed with DI water and dried with N_2_ gas. The top Au layer (2 nm Cr for adhesion, followed by 15 nm Au) was then deposited using thermal evaporation. The completed device was allowed to soak in DI water overnight before use.

### AgNPs generation

AgNPs were generated in the etalons by loading the microgels with Ag^+^ followed by their *in situ* reduction with NaBH_4_.^34–37^ Briefly, a 10 mm^2^ etalon was cut from the as-prepared larger etalon (25 × 25 mm) and was subsequently immersed in 20 mL of DI water. The solution pH was then adjusted to 8.5 with 10 mM NaOH and the etalon was allowed to incubate overnight. The etalons were then added to a 10 mL solution containing 10 mM AgNO_3_ prepared with DI water for 24 h to allow for ion-exchange between Na^+^ and Ag^+^. Then, the etalons were immersed in 20 mL of DI water overnight to remove free Ag^+^. The Ag^+^-loaded etalons were then soaked in 10 mL of an aqueous solution of NaBH_4_ (10 mM) for 30 min in an ice water bath. Finally, the AgNP-loaded etalons were immersed in DI water and stored at 4 °C.

### H_2_O_2_ sensing

The optical properties of the devices can be predicted by eqn (1):

1 *mλ* = 2*nd* cos *θ*

where *n* is the refractive index of the dielectric layer, *d* is the distance between the etalon's two Au layers, *θ* is the angle of incident light relative to the device normal, and *m* (an integer) is the order of the reflected peak.

The etalons were fixed in a specially designed stainless steel sample chamber and immersed in 30 mL pH 7.0 phosphate buffer solution (5 mM) at room temperature. The chamber was sealed with a metal cover with a port for H_2_O_2_ addition (30% w/w, 10 M) could be introduced to the solution. The center of the cover had a hole where the reflectance probe could be secured and exposed to the etalon. The light source's intensity and distance from the etalon was adjusted to result in the highest quality reflectance spectra (well defined peaks with high intensity). Spectra were recorded until they stabilized then more H_2_O_2_ was added to solution. Each experiment was repeated at least three times. To regenerate AgNPs in the etalons, a similar process to the original AgNPs generation was used. Briefly, the used etalons were immersed in 20 mL of DI water adjusted to 8.5 with 10 mM NaOH and allowed to incubate overnight. The etalons were then added to a 10 mL aqueous solution containing 10 mM AgNO_3_ for 24 h and then in DI water overnight. The Ag^+^-reloaded etalons were then soaked in 10 mL of an aqueous solution of NaBH_4_ (10 mM) for 30 min in an ice water bath. Finally, the recharged AgNP-loaded etalons were prepared and stored in DI water at 4 °C.

## Results and discussion

**Scheme 1:**
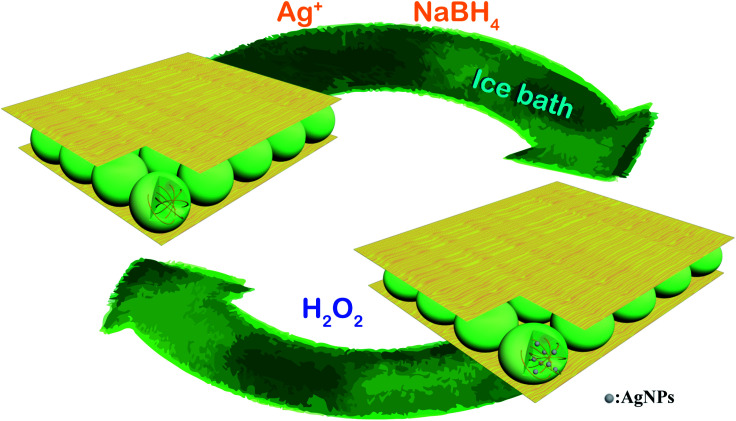
Schematic illustration of the *in situ* fabrication method of AgNPs@pNIPAm-*co*-AAc hybrid microgels-based etalon and response mechanism of the etalon.

**Fig. 1:**
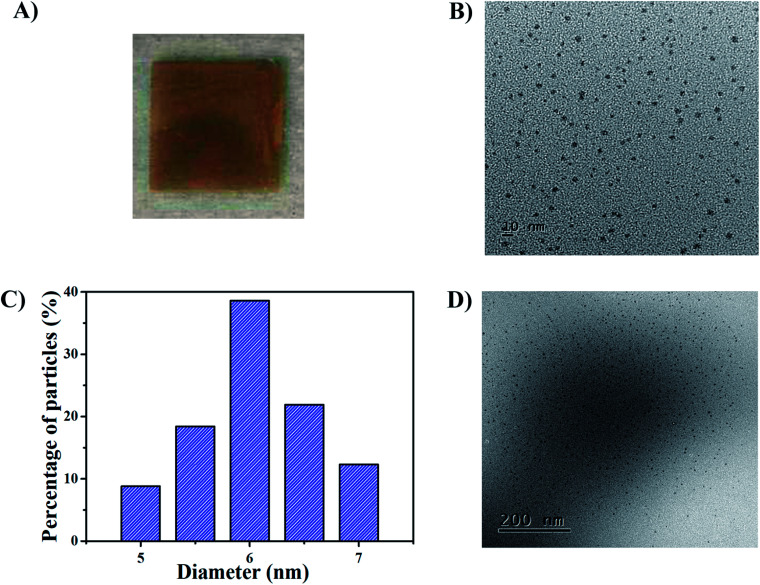
(A) Photograph of the AgNP-loaded etalon in solution at pH 7.0. (B) TEM image of AgNPs within microgels and (C) the corresponding histogram of size distribution of AgNPs. (D) TEM image of the AgNPs@pNIPAm-*co*-AAc hybrid microgels which were collected from an etalon prepared without the top gold layer (see Experimental section).

The synthetic route for generating AgNPs inside pNIPAm-*co*-AAc microgel-based etalon is shown schematically in Scheme 1. The solution pH for loading Ag^+^ was 8.5, according to our previous report.^22^ The pNIPAm-*co*-AAc microgels were composed of COOH groups that could be deprotonated by increasing the solution pH above the p*K*_a_ for AAc (4.25), which rendered them negatively charged. The negative charges in the pNIPAm-*co*-AAc microgels could increase the interaction strength between the microgels and the Ag^+^, thus leading to enrichment in the microgels. As detailed in the Experimental section above, the microgels in the etalons were then soaked in DI water to remove any unbound Ag^+^, and the Ag^+^-loaded etalon subsequently exposed to NaBH_4_ to form AgNPs. Of note, the *in situ* reduction of Ag^+^ was performed at low temperature by immersing the reaction vessel into an ice-water bath, which largely prevents the liberation of hydrogen gas.^38^ We found that the AgNP synthesis at room temperature generates hydrogen gas bubbles, due to the rapid hydrolysis of NaBH_4_, which leads to the destruction of the etalon's top Au layer, and a loss of the etalon's optical properties (Fig. S1†). As can be seen in Fig. 1A, the etalon was undamaged using the low temperature AgNP synthesis. We note that because the etalons were undamaged by this route to generating AgNPs, the AgNPs could be regenerated multiple times in the etalon for reuse. Furthermore, the AgNP synthesis at low temperature was found to be effective at narrowing the size distribution of nanoparticles, compared to what was reported previously.^39–42^ In this study, the as-synthesized AgNPs possess a rather uniform size (Fig. 1B) ranging from 5 to 7 nm (Fig. 1C) and no large diameter particles were observed, as evidenced in the representative TEM image of the hybrid microgels (Fig. 1D). Furthermore, as can be seen in Fig. S2,† characteristic binding energy peaks of Ag 3d_5/2_ and Ag 3d_3/2_ appeared at 367.9 and 373.9 eV, respectively, after the incorporation of the AgNPs. Such binding energies are between Ag^0^ (3d_5/2_: 368.2 eV and 3d_3/2_: 374.2 eV, respectively) and Ag^+^ (3d_5/2_: 367.5 eV and 3d_3/2_: 373.5 eV, respectively). The shift to lower binding energies for the AgNPs can be attributed to the interaction between oxygen and silver particles.^43^ These observations indicate the role of the microgels as microreactors, where the coordinated Ag^+^ could be steady reduced to AgNPs by NaBH_4_ in the microgel network.

**Fig. 2:**
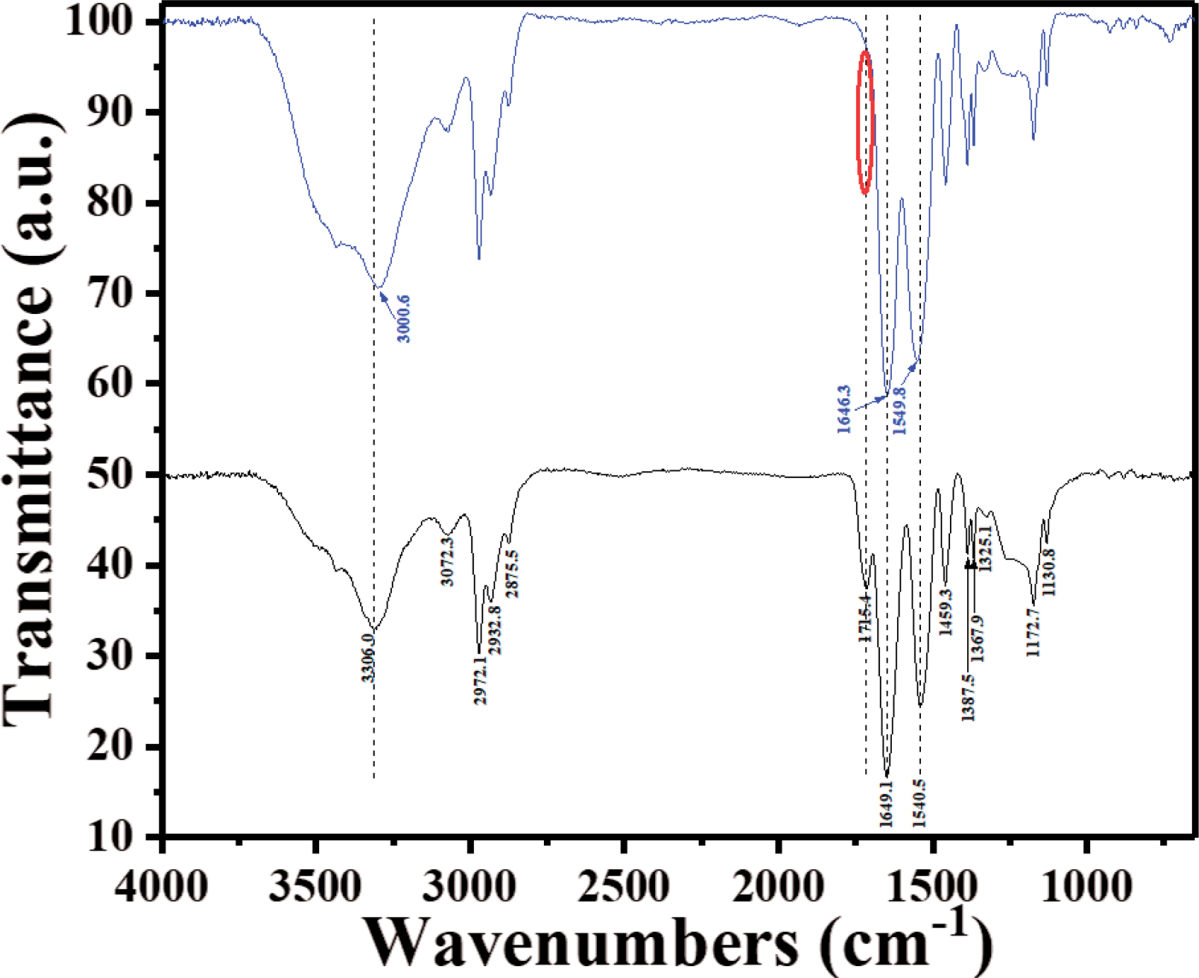
FT-IR spectra of the blank microgels (black) and the AgNPs@pNIPAm-*co*-AAc hybrid microgels (blue). The red circle shows the band where the free carboxyl group is.

The role of the pNIPAm-*co*-AAc microgels as ligands to AgNPs was then investigated by FTIR spectroscopy. As shown in Fig. 2, the pure pNIPAm-*co*-AAc microgels exhibited a series of bands centered at 3306 cm^−1^, 1715 cm^−1^, 1649 cm^−1^ and 1541 cm^−1^, which were attributed to the N–H stretching,^44^ free carboxyl group (–COOH),^45^ the amide carbonyl I bond,^46^ and the amide carbonyl II bond,^47^ respectively, in accordance with the characteristic IR absorption of the pNIPAm-*co*-AAc polymers.^41^ The incorporation of AgNPs to form the hybrid microgels was shown to lead to three major changes, as shown in Fig. 2. First, the N–H stretching peak (3306 cm^−1^) was shifted to 3300 cm^−1^; second, the free –COOH group band peak at 1715 cm^−1^ disappeared; and third, the amide carbonyl band (I: 1649 cm^−1^ and II: 1541 cm^−1^) was shifted to 1646 cm^−1^ and 1550 cm^−1^, respectively, indicating the binding interaction between AgNPs and the polymer network. This was also supported by previous reports that the coordination between metal cations and nitrogen species of pNIPAm-based microgels could lead to a shift of the carbonyl bands.^48^

Since the microgels have a high affinity for the AgNPs, we hypothesize that they act as a microgel crosslinker. That is, the binding interaction of microgels with AgNPs generates a contractive force, rendering the microgels deswollen; therefore, the microgels should swell upon removal of the AgNPs. As a result, we expect the removal of AgNPs from microgels in the etalons to yield a red-shift in the etalon's reflectance peaks, as predicted from eqn (1). AgNPs are known to decompose in the presence of H_2_O_2_ through oxidation.^22^ The proposed decomposition mechanism is showed as follows:^22^

2 Ag_*n*_ + H_2_O_2_ = Ag_*n*_/HOOH

3 Ag_*n*_/HOOH = Ag_*n*_/HOO˙

4 2Ag_*n*_/HOO˙ = 2Ag_*n*_ + 2HO˙ + O_2_

5 Ag_*n*_ + HO˙ = Ag_*n*−1_/[Ag^+^] + H_2_O

**Scheme 2:**
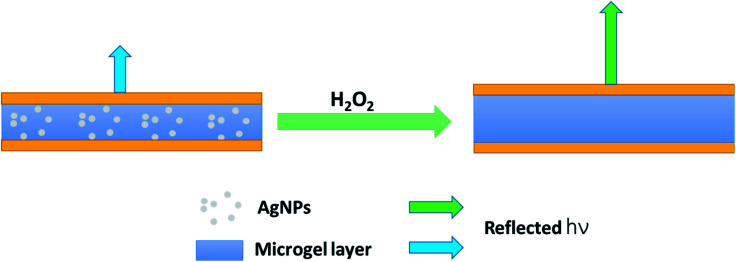
Schematic depiction of the H_2_O_2_ sensing process. (Left), light of a specific wavelength is reflected, while a different wavelength with increased intensity (indicated by the longer arrow) is reflected after the AgNPs are degraded upon H_2_O_2_ addition.

**Fig. 3:**
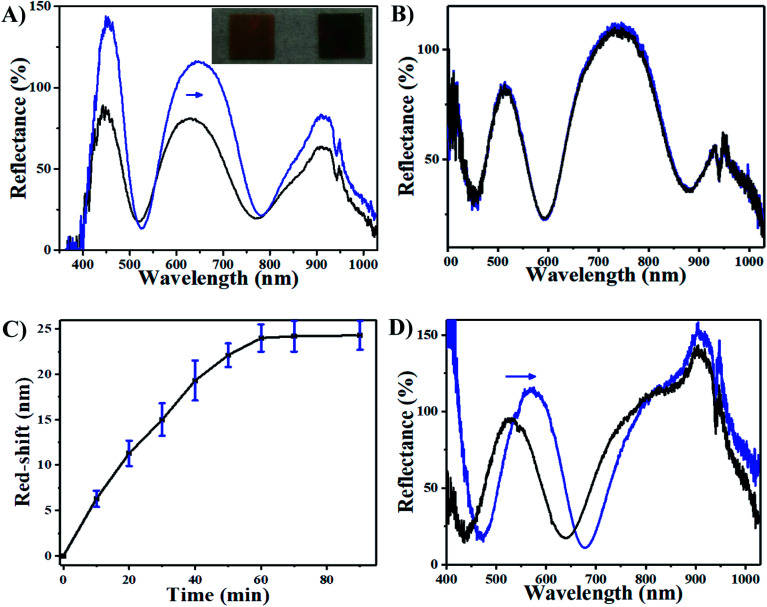
Reflectance spectra of etalon (A) with and (B) without AgNPs upon exposure to 10 mM H_2_O_2_ at pH 7.0. The inserted photograph in (A) shows the AgNPs containing etalon before and after adding 10 mM H_2_O_2_ from left to right, respectively. (C) Peak shifts as a function of reaction time after addition of 10 mM H_2_O_2_ at pH 7.0. Data are mean values ± standard deviation of three independent experiments. (D) Reflectance spectra of etalon with AgNPs upon exposure to 10 mM H_2_O_2_ at pH 3.0. The blue arrows show the direction of peak shift.

We thus hypothesize that the introduction of H_2_O_2_ can trigger a red-shift of the reflectance peaks from the AgNP-loaded etalons. As can be seen in Fig. 3A, a characteristic multipeak reflectance spectrum of the AgNP-etalon was observed and it shows three reflectance peaks at 475 nm, 635 nm and 910 nm. The most significant peak (*i.e.*, 636 nm) was selected in the following experiments for analysis. It was observed that the peak red-shifted 24 nm in the presence of 10 mM H_2_O_2_, while no changes were observed by adding the same amount of H_2_O_2_ to an etalon without AgNPs (Fig. 3B), indicating that the shift could be a result of the presence of the AgNPs. Furthermore, we observed a change in the intensity of the reflectance peaks in response to H_2_O_2_, which is not typically observed from the microgel-based etalons.^4,49^ AgNPs are also well known to absorb visible light due to the excitation of plasmons.^50^ With the etalons here, we also found that the intensity of reflected light increased from 75% to 120%, possibly due to the reduction of AgNP light absorption. Thus, more photons could make it back to the reflectance probe, as opposed to being absorbed by the AgNPs. In addition, the intensity of the peak valley (∼25%), almost remained unchanged. This increase of the intensity ratio of peak to valley improved contrast, allowing for facile visual characterization. Specifically, the addition of H_2_O_2_ led to the color change of the AgNP-etalon from brassy yellow to green (Fig. 3A inset). Therefore, sensing H_2_O_2_ by monitoring two parameters, *i.e.*, red-shifted reflectance peak and increased intensity, can be attributed to the swelling of the microgel layer and the reduction of the AgNP absorption, respectively, as a result of decomposition of the loaded AgNPs. The H_2_O_2_ sensing mechanism is depicted in Scheme 2.

**Fig. 4:**
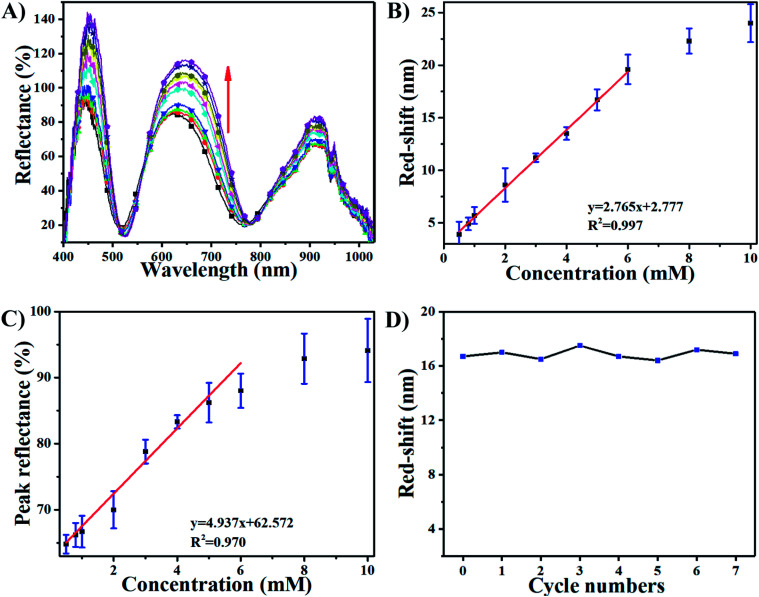
(A) Reflectance spectra of AgNPs-loaded etalon upon exposure to various concentrations of H_2_O_2_ at pH 7.0 from 0.5 (bottom) to 10 mM (top); the direction of the peak shift is indicated by the red arrow. (B) Peak shifts as a function of H_2_O_2_ concentration. (C) Peak reflectance as a function of H_2_O_2_ concentration. (D) The cycles of the red shifts of etalon though the “consuming-recharging” of AgNPs at pH 7.0 (H_2_O_2_ concentration: 5 mM). Data are mean ± standard deviation of three independent experiments.

The sensing performance of this device was further investigated by exposing it to 10 mM H_2_O_2_. Fig. 3C shows that the response of the AgNP-etalon to H_2_O_2_ was complete in ∼1 h. We note that the responsivity of this device towards H_2_O_2_ was enhanced (Fig. 3D) in pH 3.0 buffer solution, possibly due to the reduction of electrostatic repulsions of protonated intra-microgels (p*K*_a_ of AAc: 4.25), which suppressed the microgel deswelling. Next, we determined the ability of the AgNP-loaded etalons to quantify H_2_O_2_ concentration in solution. As can be seen in Fig. 4A, as the concentration of H_2_O_2_ increased from 0.5 mM to 10 mM, the selected reflectance peak of the AgNP-etalon correspondingly red-shifted, exhibiting a good linear relationship within the concentration ranging from 0.5 mM to 6 mM (*R*^2^ = 0.9943) (Fig. 4B), which is comparable to previous methods of H_2_O_2_ detection (Table S1†). Also, the reflectance intensity of this device increased in response to escalated concentrations of H_2_O_2_ (Fig. 4C).

As the exposure of the etalons to H_2_O_2_ caused the dissolution of the AgNPs, their regeneration in the etalons was also investigated. Specifically, addition of 5 mM H_2_O_2_ caused 16 nm red-shift of the reflectance peak of the device (Fig. 4A and D) and a dramatic decrease in the amount of AgNPs in the microgels, as revealed by the representative TEM images in Fig. S3.† Due to the etalon structure remaining intact, we reason that AgNPs could be regenerated in the microgels and the etalons reused. To show this, we reloaded the microgels with Ag^+^ and carried out the NaBH_4_ reduction, as described in Experiment section above. After incorporating AgNPs in the etalon again, a similar response of the etalon to H_2_O_2_ can be fully recovered, as can be seen in Fig. 4D. Furthermore, the AgNP regeneration could be repeated at least 7 times without losing the performance efficiency of this device, as shown in Fig. 4D.

## Conclusion

In summary, we reported an approach for the *in situ* generation of AgNPs inside pNIPAm-*co*-AAc microgels that were the responsive component of an etalon. We characterized the synthesized AgNPs-loaded microgels within the etalon, and demonstrated that the incorporation of the AgNPs allowed the etalons to respond to H_2_O_2_ in solution by changing the wavelength of the reflected light and the reflected light intensity. We attributed this dual response towards H_2_O_2_ to the oxidative decomposition of the AgNPs within the microgels, enabling the microgels to swell and the light that was absorbed by the AgNPs to be reflected. Finally, we demonstrated that the functional etalons could be reused multiple times through repeatedly “consuming and recharging” the AgNPs. While this study was only done with H_2_O_2_ as a proof of concept, it is a promising approach to extend the versatility of etalons to sense species of broad relevance simply by modification with nanomaterials, enriching the polymer-based photonic sensor portfolio. This work has also demonstrated that the dual signal sensing can be a potential route to improved sensor performance, *i.e.*, enhanced selectivity and accuracy.

## Conflicts of interest

There are no conflicts to declare.

## Supplementary Material

RA-008-C8RA02215A-s001
