# Supplementary material for: Silver nanoparticle-loaded microgel-based etalons for H2O2 sensing
Source: RSC Adv. 2018 Apr 24;8(28):15567–74. doi: 10.1039/c8ra02215a (PMC9080173; doi:10.1039/c8ra02215a)
Supplement: RA-008-C8RA02215A-s001 [file RA-008-C8RA02215A-s001.pdf]

Electronic Supplementary Material (ESI) for RSC Advances.
This journal is © The Royal Society of Chemistry 2018

Supporting Information

Silver Nanoparticle-Loaded Microgel-Based Etalons for H_2O_2 Sensing

Tong Shu,[a,b] Qiming Shen,[b] Yu Wan,[b] Wei Zhang,[b] Lei Su,[a] Xueji Zhang[a] and Michael J. Serpe*[b]

[a] Beijing Advanced Innovation Center of Materials Genome Engineering, Beijing Key Laboratory for Bioengineering and Sensing Technology, Research Center for Bioengineering and Sensing Technology, University of Science and Technology Beijing, Beijing 100083, P. R. China;

[b]Department of Chemistry, University of Alberta, Edmonton, Alberta, Canada.

Department of Chemistry, University of Alberta, Edmonton, Alberta, Canada T6G 2G2. E-mail: michael.serpe@ualberta.ca;

Fax: +1 780 492 8231; Tel: +1 780 492 5778

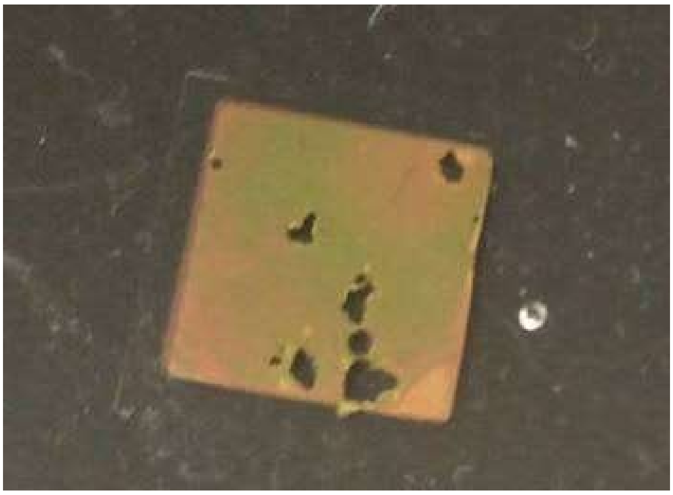

Figure S1. Photograph of an etalon made via the in situ synthesis of AgNPs in the etalon at room temperature.

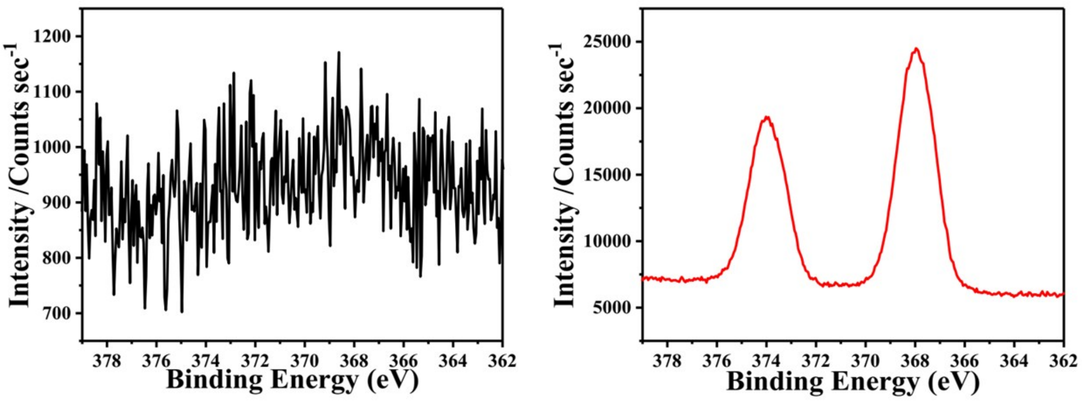

Figure S2. X-ray photoelectron spectroscopy spectrum of pNIPAm-co-AAc microgels (left) before and after incorporating the AgNPs (right).

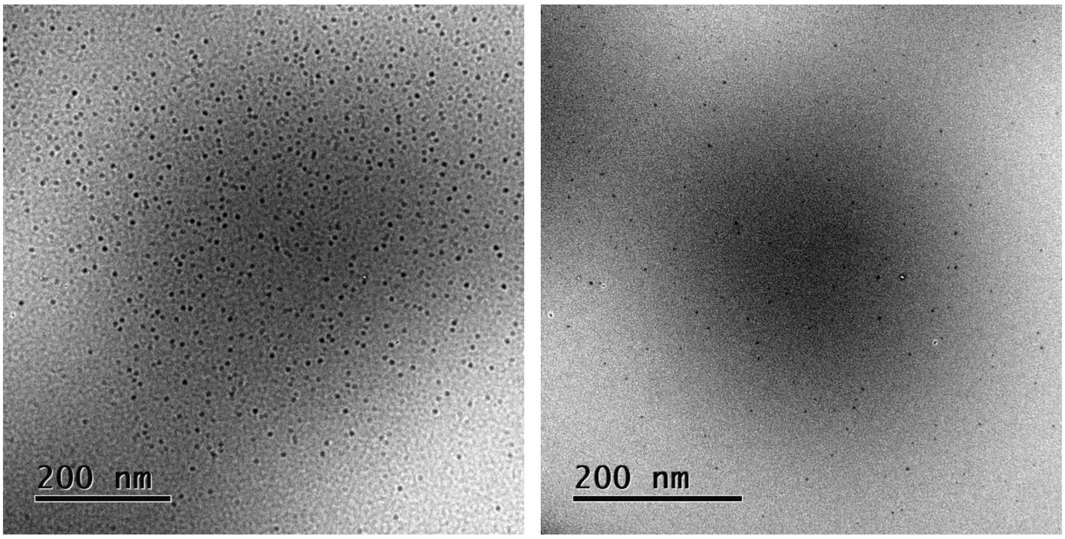

Figure S3. TEM images of the AgNPs incorporated microgels (left) before and (right) after treating with 6 mM H_2O_2.

Table S1. A partial list of H_2O_2 sensors

Materials	Linear range (mM)	Reference
Graphene wrapped Cu_2O nanocube	0.3-3	[1]
Fe_2O_3/graphite	0.2-5.5	[2]
rGO/Fe_3O_4	0.1-6	[3]
Graphene nanosheets	1-20	[4]
boron-doped graphene nanosheets	1-20	[4]
Ferrocene-Modified Microgels	0.6-35	[5]
AgNPs-incorporated microgels	0.5-6	This work

References

1 M. Liu, R. Liu and W. Chen, *Biosens. Bioelectron.*, 2013, **45**, 206.

2 B. Šljukić, C. E. Banks, A. Crossley and R. G. Compton, *Electroanal.*, 2006, **18**, 1757.

3 Y. P. Ye, T. Kong, X. F. Yu, Y. K. Wu, K. Zhang and X. P. Wang, *Talanta*, 2012, **89**, 417.

4 M. H. Yeh, Y. S. Li, G. L. Chen, L. Y. Lin, T. J. Li, H. M. Chuang, C. Y. Hsieh, S. C. Lo, W. H. Chiang and K. C. Hoa, *Electrochim. Acta*, 2015, **172**, 52.

5 Q. M. Zhang, D. Berg, J. Duan, S. M. Mugo and M. J. Serpe, *ACS Appl. Mater. Inter.*, 2016, **8**, 27264.
